# The Advantages of Demographic Change after the Wave: Fewer and Older, but Healthier, Greener, and More Productive?

**DOI:** 10.1371/journal.pone.0108501

**Published:** 2014-09-24

**Authors:** Fanny Kluge, Emilio Zagheni, Elke Loichinger, Tobias Vogt

**Affiliations:** 1 Laboratory of Survival and Longevity, Max Planck Institute for Demographic Research, Rostock, Germany; 2 Department of Sociology, University of Washington, Seattle, Washington, United States of America; 3 International Institute for Applied Systems Analysis, Laxenburg, Austria; 4 Research Institute for Human Capital and Development, Vienna University of Economics and Business, Vienna, Austria; City University of New York (CUNY), United States of America

## Abstract

Population aging is an inevitable global demographic process. Most of the literature on the consequences of demographic change focuses on the economic and societal challenges that we will face as people live longer and have fewer children. In this paper, we (a) briefly describe key trends and projections of the magnitude and speed of population aging; (b) discuss the economic, social, and environmental consequences of population aging; and (c) investigate some of the opportunities that aging societies create. We use Germany as a case study. However, the general insights that we obtain can be generalized to other developed countries. We argue that there may be positive unintended side effects of population aging that can be leveraged to address pressing environmental problems and issues of gender inequality and intergenerational ties.

## Introduction

Countries around the globe are experiencing population aging. In developed societies, life expectancy has risen at a steady pace of about three months per year, and this trend is not expected to end [1]. At the same time, fertility fell considerably in almost all countries around the world. In Europe, three-quarters of the population live in countries with fertility below the replacement level [2]. Increasing longevity and low birth rates inevitably result in major changes in the observed age structure, with consequences for societal arrangements. The projected changes will affect numerous areas of life, such as family formation, labor market arrangements, the sustainability of public finances, and the environment.

Economic research has so far primarily stressed the negative effects of increasing life expectancy and declining fertility, such as the burden of increasing dependency ratios [3], [4], the dramatic effects on the labor market [5]–[8], and the effects of global aging on the macroeconomy [9], [10]. Furthermore, a big branch of the literature focuses on the impact of population aging on the pillars of social security. According to numerous studies, health expenditures are going to increase [11]–[14], as are expenditures for long-term care [15], [16]. Conversely, other studies find that population aging contributes little to the escalation of health care costs [17], [18]. The increasing share of public pension expenditures of government budgets is also of great concern [19]–[22]. The observed and expected changes in the demographic composition are projected to cause different problems in different countries, depending mainly on the degree of reliance of the citizens on the public sector in old age and the generosity of public programs.

For most European countries, the most dramatic changes are expected to occur over the next three decades, during which a decreasing number of producers will have to care for an increasing number of consumers. After this transitional period, the populations of these countries will, on average, be older and smaller; and the costs will be lower as smaller cohorts start entering retirement age. Therefore, while it is certainly true that the current and expected changes in age structure will have a negative impact on, for example, public finances, in the medium term, we expect to see much milder consequences in the long run. In fact, there are a number of ways in which population aging could represent an opportunity. We investigate five areas that can illustrate possible unintended positive consequences of population aging in the long run. We focus on the educational composition of the labor force, CO_2_ emissions in aging societies, intergenerational transfers in the form of bequests, developments in care need and healthy life expectancy, and the share of the lifetime spent working. These five examples demonstrate the potential positive long-term effects that are either directly or indirectly caused by population aging. Examples of direct consequences are lower CO_2_ emissions due to changing consumption patterns over the life cycle, and more balanced intergenerational transfers as the upward public transfers for public pensions are compensated for through higher inter-vivo transfers and bequests due to the lower number of siblings. An example of indirect consequences of population aging is the likely change in composition of the labor force due to the expected changes in the education structure.

Our study demonstrates the positive effects if the current conditions prevail. However, this is not necessarily the case: There will be some degree of behavioral adaptations of individuals, i.e., the findings are subject to change related to how far individuals in the future make decisions that deviate from the currently observed patterns. While it would be possible to speculate about the different coping strategies that may be implemented in the future, making such predictions is beyond the scope of this article. However, our results will give some indication of the potential magnitudes of expected effects. Hence, we intend to offer some basis for discussion about the possible advantages of population aging and how key age-dependent patterns could lead to changes that will entail important societal adjustments.

We mainly focus on Germany, not only because the country is a forerunner in terms of aging, but also because it has a very high level of public transfers and a huge industrial sector. These characteristics make Germany highly suitable for the envisaged analysis. The article is structured as follows: We first provide an overview of the population aging process in Germany. We then present five illustrative examples of areas in which aging might have advantageous side effects. In the remainder of the paper, we show why these developments can be important for policy-makers, and provide a concluding discussion.

### Aging in Germany

**Figure 1:**
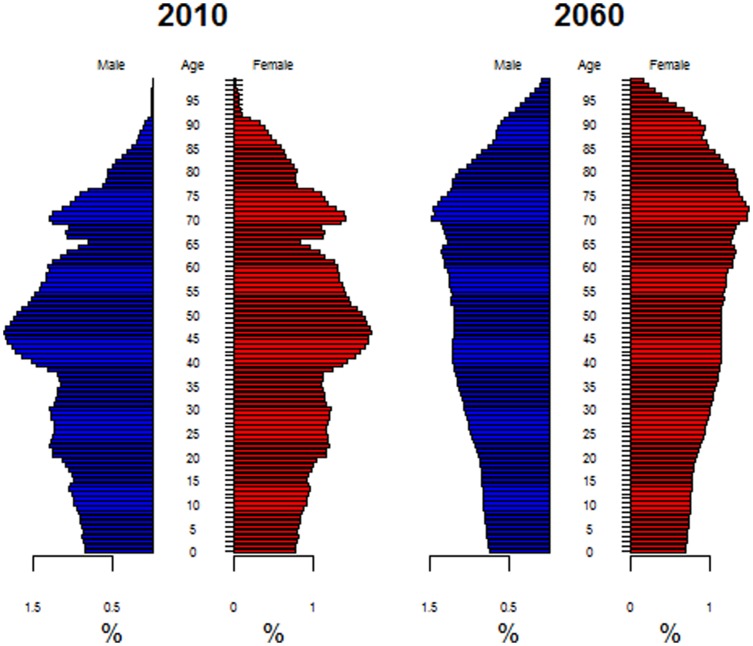
Population age distribution, Germany 2010 (left) and 2060 (right). Source: Statistisches Bundesamt (2012).

We begin our analysis by presenting some descriptive figures on population aging in Germany. Our intention is to show that major demographic changes have happened in the past, and that ongoing changes are expected to shape the future until 2040. The main reason for these developments is the fact that the baby boomer cohorts are reaching retirement age. When this influential transition phase – which is expected to put pressure on social security programs and create more general sustainability issues – has passed, a more “advantageous” population age distribution can be expected. Figure 1 compares the population age distributions for Germany in 2010 and 2060. While today individuals of working age represent the majority of the population, these cohorts will grow old in the coming decades. Thereafter, we can expect to see an older but more evenly distributed age structure.

Germany is at an advanced stage in the demographic transition for two main reasons: an early fertility decline and a rapidly increasing life expectancy. The TFR had already fallen far below the replacement level in the early 1970s and even today remains at around 1.4. At the same time, life expectancy is expected to increase further. In the medium-scenario UN projection, women are predicted to gain another 10 years of life expectancy over the course of the century. By 2060, female life expectancy at birth is expected to reach 90 years [23].

Already today, Germany’s population has the second highest median age worldwide (44.3 years) only topped by Japan’s median age of 44.9 years [24]. This makes Germany a forerunner in terms of aging, with a very steep rise in the old-age dependency ratio set to occur between 2020 and 2040. The economic support ratio shows an even more refined picture, as it considers the real dependencies, and is, for example, used to calculate demographic dividends [25]. In this case SR(t) = L(t)/N(t) where L(t) is the population weighted by age-specific variation in productivity and N(t) is the population weighted by the variation by age in consumption. To calculate the support ratio, we use data on age-specific consumption and income patterns from the National Transfer Accounts project (see www.ntaccounts.org) to estimate the real consumption and income values for the population over the coming decades. The support ratio shows that, currently, about 0.8 producers take care of one consumer. In the decades to come, this ratio will fall to 0.6. Thus, Germany is an important case to study, as it is among the first of the countries that will experience a demographic future which almost all of the industrialized countries will eventually face.

As Germany likely faces a continuously low level of TFR and parallel increases in life expectancy, the aging process will occur at a rapid pace, which as stated earlier will lead to sustainability problems. While this is certainly the case in specific areas and for some transitional decades, we can expect the demographic changes to be moderate after 2050. We have identified important areas in which population aging can be even advantageous. These areas will be presented in the remaining sections of the paper.

## Methods

We use various methods to show the likely advantageous effects of population aging in selected areas, but we will not describe these methods in detail here. Instead, at the beginning of each section, we will briefly present the data and methods used. The overall approach for all of the sections is comparable: we will show the underlying age patterns for each variable of interest, such as the CO2 emissions or the education-specific labor force participation level. This is intended to provide information on how age-sensitive our variables are. We will then project the information. Our focus is on a point in time relatively far in the future, when the demographic turmoil is likely to be over, and a smaller population with a less unbalanced age structure is anticipated. Our analysis mainly investigates what will happen if the current conditions prevail. We use age profiles of the monetary production and consumption, environmental conditions, the labor market, health-related conditions and time use variables observed today, and combine these with medium-term population forecasts, to show what happens to the results on the macro level if a different population structure were assumed. This basic forecast can give us important insights into how demography will alter the variables on the population level; and whether we will encounter an improvement in, for example, CO_2_ emissions after 2050, if only because having an older population is, on average, associated with lower consumption of energy-intensive goods.

## Results

### More productive? – Demographic change and the labor force

**Figure 2:**
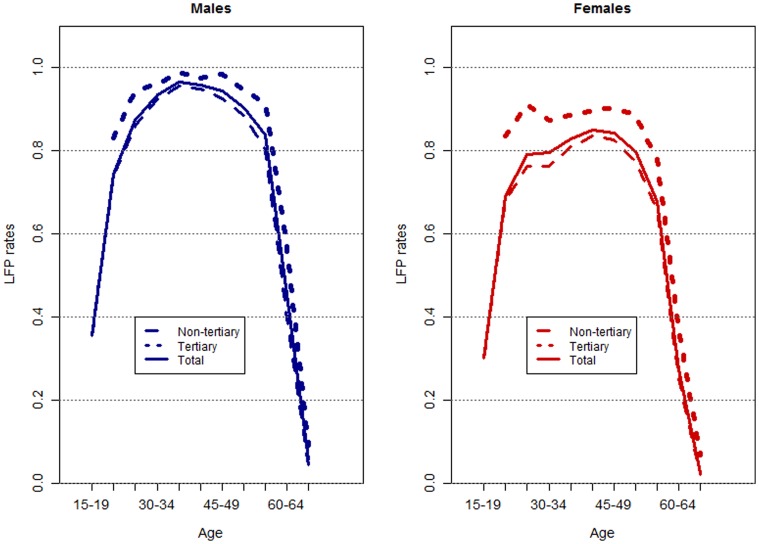
Labor force participation rates in Germany, by age, sex, and highest level of educational attainment, 2008. Source: EU LFS, own calculations.

Economic activity levels in Germany vary between age groups and between men and women, and they show the typical inverted u-shape: the level is higher for middle-aged individuals than for younger and older people, and men are more likely to participate in market work than women (the “total” line in Figure 2). In addition to these known differences, there are also noticeable differences in labor force participation levels – our measure of economic activity – by individuals’ highest levels of educational attainment. We created two distinct education categories: one that includes everyone with a tertiary degree (equivalent to ISCED level 5 or 6), and one that includes everyone who has a lower level of educational attainment (ISCED level 1 through 4). Figure 2 shows labor force participation levels in Germany across age, sex, and education for the year 2008, based on the European Labor Force Survey, EU LFS [26]. The year was deliberately chosen to avoid including the impact of the financial crisis. Labor force participation is defined according to the definition of the ILO, which means it includes the employed as well as the unemployed. The labor force participation rate is defined as the sum of employed and unemployed persons divided by the total number of people in the respective age, sex, and education category. The higher the level of education, the higher the level of labor force participation, and the smaller the differences between men and women. The total size of the German labor force was about 42.6 million in 2008, and one-quarter of the individuals in the labor force had a tertiary degree. This share was slightly higher among men than among women; or 27% and 23%, respectively.

The fact that economic activity levels not only show distinct patterns by age and sex, but also by education, plays a crucial role when it comes to assessing the potential impact of changes in the size and structure of the labor force on economic growth. The consequences of population aging are only to a certain degree determined by a population’s age structure. Labor productivity is not uniform, but varies along several dimensions, with one of them being the educational attainment levels of workers [10]. It might very well be the case that a smaller and older but – in terms of human capital – better equipped and more productive labor force can compensate for some of the expected declines in economic growth [27], [28]. However, it is not the goal of this exercise to quantify these growth effects. Instead, we would like to show the potential changes in the composition of the German labor force that go beyond the usual projections by age and sex.

Before we can project the characteristics of the labor force in terms of age, sex, and education, we need not only the participation profiles along these three dimensions, but also the corresponding population projections. Using the multi-state cohort-component method, the German population was projected from 2008 to 2053 [29]. The starting population distribution in 2008 was calculated from the EU LFS. The specifications for future fertility, mortality, migration, and education transitions are the same as those presented in [30]; with the only difference being that the assumptions about the future development of fertility, mortality, and migration have been updated using the 2011 Version of the World Population Prospects [23]. The education transitions came from the GET (Global Education Trend) scenario, which projected future transitions based on the development of historical global patterns of educational expansion. On the basis of these assumptions, our analysis showed that not only will larger shares of the younger cohorts attain tertiary education (33% of 25–29-year-olds in 2053, compared to 19% in 2008), but the share of the population with higher levels of education will increase among the age groups 50 and older, as better educated cohorts will replace cohorts with lower levels of educational attainment (“educational upgrading”). This means that the share of Germans aged 50 and older with a tertiary degree was 21% in 2008, and is projected to be 34% in 2053.

Keeping the labor force participation profiles fixed at the levels observed in 2008 and combining them with the education-specific population projections leads to an absolute labor force size of 29.6 million in 2053, which represents a reduction of almost one-third compared to 2008. This projected decline in the number of economically active individuals in Germany, based purely on changes in the demographic structure, has appeared in previous research [31]. However, by including the educational dimension, we can also offer some estimates regarding the educational composition of the future labor force: the share of the labor force with tertiary education was 25% in 2008, but it is projected to rise to 33% in 2033, and to increase further to 41% in 2053 (compare Table 1). Since participation is held constant, this change is solely due to the changes in the composition of the German population described above. Table 1 shows that this change is expected to happen among all of the age groups, although to varying degrees.

**Table 1:** Share of the German labor force with tertiary education, 2008 and 2053 (constant participation scenario).

Age-group	2008	2053
**25–29**	21%	36%
**30–49**	29%	49%
**50+**	30%	44%
**Overall (25+)**	28%	46%

Source: Loichinger (2012), own calculations.

Based on the observation of past trends in Germany and other aging countries, increases in the labor force participation rates of women and of people over age 50 are likely. In order to estimate what the consequences of these anticipated future developments would be, we calculated an additional scenario in which we assumed that participation would, by 2053, reach the levels that were observed in Sweden in 2008. Sweden is often cited as a role model country when it comes to equality in economic activity levels among men and women and to the labor force participation of workers over age 50. For example, in 2008 the labor force participation rate of 60–64-year-olds was 61% in Sweden, but only 36% in Germany.

Under the assumptions of this second scenario, we can see that the total size of the labor force is still set to decline, but only to 33.7 million: this represents a reduction of 20%, rather than the reduction of over 30% that is anticipated to occur in the constant scenario. Since the total population of Germany is projected to decline as well – from 83 million in 2008 to roughly 70 million in 2053– the support ratio will only decline significantly under the first scenario: from 0.51 in 2008 to 0.42 in 2053. The support ratio here is defined as the ratio of persons in the labor force to those not in the labor force, including the population below age 15. Under the assumption of Swedish participation rates, the support ratio in 2053 would still be 0.49. In terms of the educational composition of the future labor force, the two scenarios show only very minor differences.

### Greener? – Demographic change and the environment

Demographic change has important consequences for the environment and for carbon emissions. In the literature that dates back to the IPAT equation [32], population size was the demographic factor that received the most attention. More recently, the study of the role of population composition has become more prominent [33].

Population aging is a process that entails substantial changes in the population age structure. It has been shown that the profile of per capita CO_2_ emissions by age has an inverted-U shape [34]. As individuals enter adulthood, they tend to consume and travel more, which in turn leads to a higher level of emissions. This trend continues well into adulthood, as older people have larger incomes and larger houses and cars. Emissions then decline when individuals retire and travel less. At an aggregate level, the inverted-U profile of emissions over the life-cycle means that a changing age structure may generate considerable reductions in CO_2_ emissions, all else being constant.

**Figure 3:**
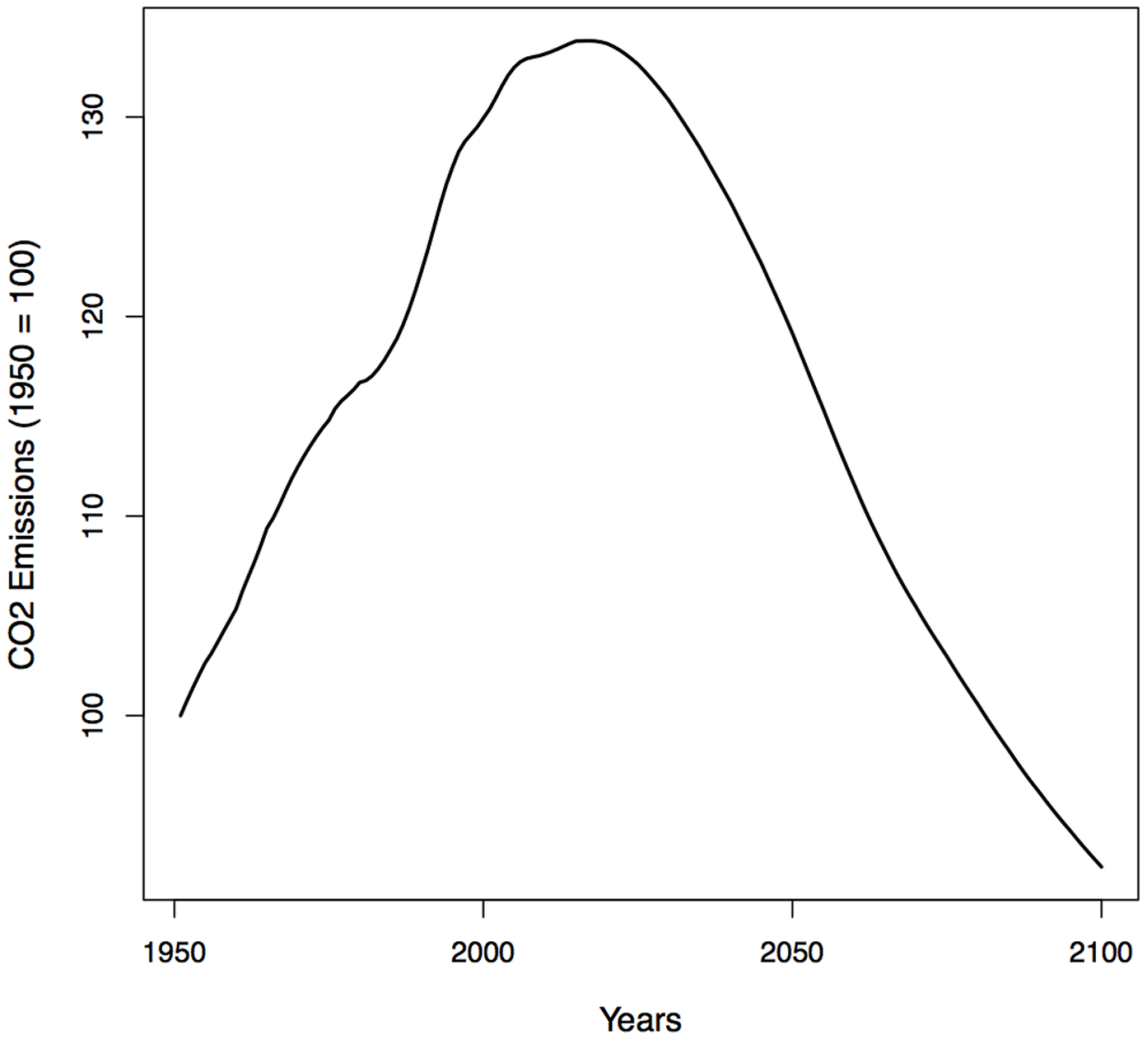
Relative change in CO_2_ emissions attributable to changes in population size and age structure affecting the sum of age-specific consumption patterns, Germany, 1950–2100. Source: Zagheni (2011), UN population projections, own calculations.

Figure 3 shows the trend in emissions for Germany, obtained by multiplying the age-specific per-capita profile in Zagheni [34] and UN population projections [24]. The profile of emissions over the life course is kept constant: changes in emissions are driven by changes in population size and age structure. In our stylized model, we assume that the relative age-profile of emissions for Germany is qualitatively similar to the one of the US and other developed countries. Under that assumption, we observe that population aging initially tends to increase emissions as a growing number of people pass through the ages at which emissions peak. We expect that the effect of changing population size and age structure may contribute to an increase in emissions of more than 30% over several decades, from 1950 to 2020. But in the long run, as the proportion of people older than age 80 continues to increase and the population size shrinks, emissions would decrease and reach pre-1950 levels, under the assumption that the age-specific behavioral contribution to emissions would not change.

The combined effects of a reduced population size and a more favorable age structure (in terms of carbon emissions) are likely to generate substantial reductions in CO_2_ emissions. These gains will not happen in isolation. New lifestyles, increased levels of wealth, and the development of new technologies will interact with the changing demographic landscape. However, this is a case in which population aging could be appropriately leveraged to transform a challenge into an opportunity.

### Richer? – Demographic change and intergenerational transfers

The impact of population aging on intergenerational transfers has been studied extensively (e.g., [35]). However, the effects of demographic change on the size and timing of bequests over the life course have not received as much attention.

The relationship between demography and bequests is complex, and is mediated by a number of economic variables, like saving rates and wealth accumulation. However, the demographic forces that shape intergenerational transfers in the form of bequests are relatively simple.

Increasing longevity and fertility postponement affect, in opposite directions, the points in time over the life course when people receive bequests. Increasing life expectancy means that, all else being constant, people will experience the deaths of their family members later in life. Thus, on average, individuals will receive bequests later in life. Fertility postponement acts in the opposite direction. Later childbearing means that, all else being equal, people in the same age group will have, on average, older parents and grandparents. In the US, life table calculations and empirical evidence from the Panel Study of Income Dynamics (PSID) indicate that the rapid increase in the mean age at childbearing has more than counteracted gains in life expectancy in recent years [36].

Lower fertility means that bequests are shared among a smaller group of people; thus the per capita amounts that individuals receive should be larger. As fertility has reached below-replacement levels, we expect that the per capita amounts of bequests people receive will stabilize.

In order to gain insight into the relative importance of changes in life expectancy and the postponement of childbearing in Germany, we evaluated trends in the life expectancy at the mean age at childbearing. This figure is a proxy for the number of years a surviving woman would expect to live with her mother alive. It can also be thought of as the average age at which a woman would experience the death of her mother, conditional on the woman’s survival. Trends in the age at the mother’s death can be seen as indicative of general trends in the age at the receipt of bequests.

**Figure 4:**
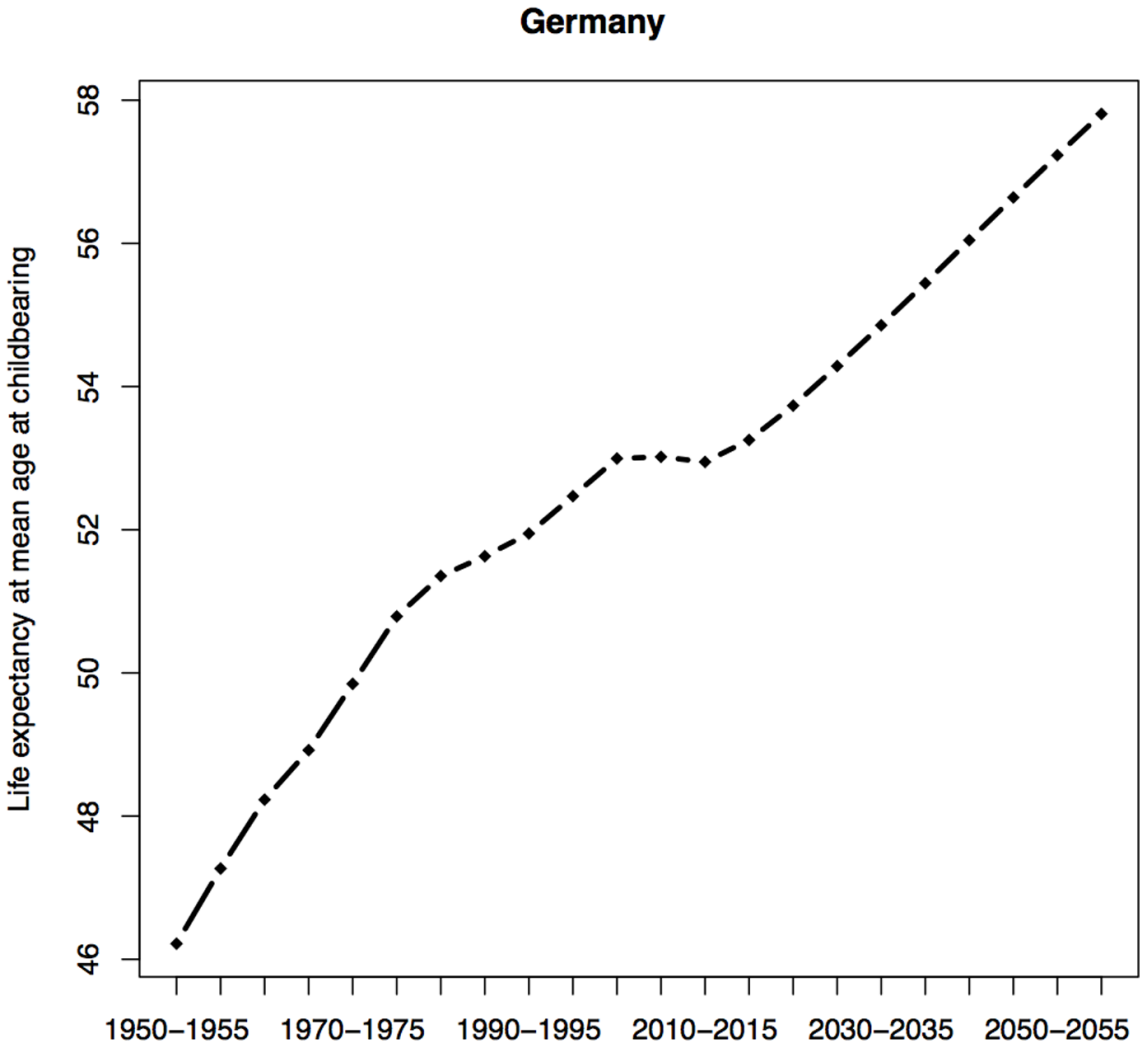
Estimates and projections of female life expectancy at the mean age at childbearing in Germany for the period 1950–2060. Source: Own calculations using demographic rates from the UN World Population Prospects: the 2012 Revision.

Figure 4 shows trends in female life expectancy at the mean age at childbearing for Germany. Overall, the general trend indicates that people inherit wealth, on average, at a later stage of their life course. The general increase in life expectancy at the mean age at childbearing seems to have stalled during the past decade, as fertility postponement counteracted gains in life expectancy. However, in the coming decades, we expect to see that the effect of continued gains in life expectancy will prevail. Assuming that people do not dis-save during retirement and that fertility remains low, gains in life expectancy, coupled with the smaller size of younger generations, may also translate into larger per capita inheritance.

As the age at which people experience the death of a family member is expected to increase over time, individuals will receive bequests, on average, when they are older; i.e., after they have established themselves professionally and are potentially close to retirement. Bequests may thus act as a form of equalizer. The family members who stand to benefit the most from the bequests–albeit indirectly–could be the grandchildren, who may receive financial help from their parents as they attend college or form a family.

An increase in the age at which bequests are received is not necessarily advantageous or disadvantageous. However, it can be seen as an opportunity in an aging society. Appropriate policies regarding inheritances could be designed to reduce inequality and favor intergenerational transfers to those who are at a stage of their life-cycle in which they require more resources.

### Healthier? – Demographic change and healthy life expectancy

The projected increases in longevity in the coming decades are substantial. In Germany, life expectancy at birth for females will rise from 83 years currently to around 90 years by 2050. In the past, gains of roughly 2.5 years were added every 10 years. These projected changes in longevity raise the question of whether the lifetime added will be spent in good health. There are several studies on current and past trends in health expectancies, including disability-free and active life expectancy [37]. They have shown improvements in health expectancies for Germany and other developed countries as measured by different indicators of limitations in the activities of daily living or of subjective health perceptions [38]–[41]. However, other studies have found that these gains in disability-free life expectancy have been accompanied by an increase in the prevalence of chronic diseases [42], [43].

Surprisingly, there are relatively few analyses of future health expectancy trends in Germany. These trends are mainly considered in studies on future expenditures on health care and long-term care in aging welfare states [11], [13], [15], [16]. Increasingly, studies have found indications that long-term care expenditures tend to be concentrated at the end of life, during the two years prior to death [44], [45]. This suggests that the increases in life expectancy are not necessarily related to soaring health care costs. However, the question remains if Germans enjoy not just longer lives, but healthier lives as well. In an effort to answer this question, we have forecasted age-and sex-specific trends in self-perceived health and the care needs of household members.

The analysis is based on micro-level data collected in the German Socio-Economic Panel Study (SOEP). The SOEP is a representative household panel study that reaches back to 1984, and interviews all of the adult household members over age 17 on an annual basis. In the first wave, 12,000 respondents were interviewed. Subsets for foreigners, eastern Germans (in 1990), and immigrants were added subsequently; and in 1998, 2006, and 2009, “refreshment” samples were included. The participating households are followed over time. The SOEP contains information about each individual respondent, including his or her working history, income parameters, and household structure. Detailed descriptions of the panel design and description of the data have been provided elsewhere [33], [36].

The SOEP data are organized into different datasets. For our analysis, we matched the data elicited in individual-level questionnaires with biographic data and health and disability information. We chose the question “How would you describe your health at present?” as an indicator for self-perceived health status; and the question “Is there anyone in your household who is receiving care because of old age or health reasons?” as an indicator for care need among the elderly members of the household. This question reflects the general need of care among older family members and spouses and, indirectly, at which ages this need is expressed for the first time. The self-perceived health question was asked between 1992 and 2011, and had five possible answers: “very good”, “good”, “satisfactory”, “poor” and “very poor”. We dichotomized this variable with the first three answers, which meant that the respondent was healthy. The care question was asked between 1984 and 2011, and had only two possible answers: “yes” and “no”. Unfortunately, the SOEP does not contain information on elderly living in residential homes for the period of observation. This leads to an underrepresentation of individuals receiving care. However, we base our estimation on onset of care need rather than the quantity of care needed. We assume that the onset of care need does not necessarily differ between individuals receiving care at home or in residences and that family members may move to residences only when they expect to need care. Moreover, spouses may care for each other at home. We used this information to calculate the sex-specific mean age at which good health/bad health and no need for care/need for care was reported. In a second step, we forecasted these trends using a random walk with drift.

**Figure 5:**
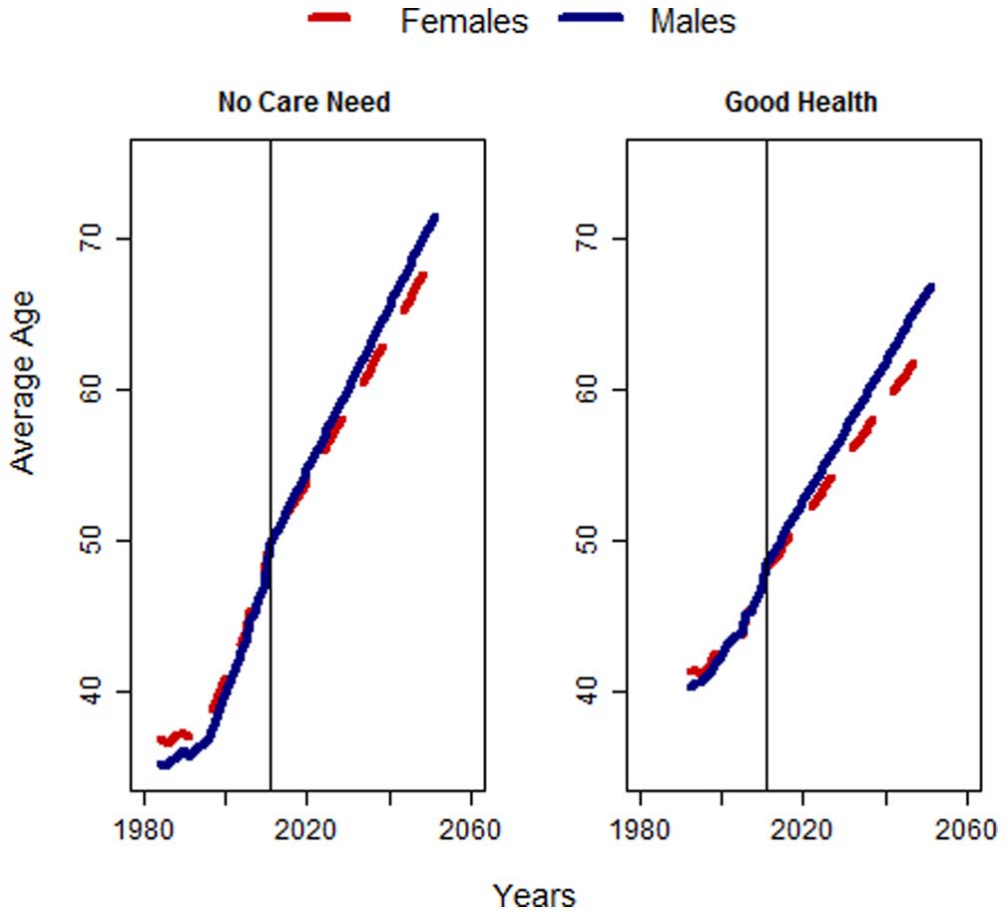
Projected average age until no care need is reported by any elderly household member (left) and good self-perceived health (right), men and women, Germany 1984–2050. Source: GSOEP 1984–2011, own calculations.

Our results confirm the findings from the literature. Between 1984 and 2011, the average age at which need for care is reported for the first time rose by 13 years, from 36 to 49 years, among women. Assuming that the pace of improvement is similar in the future, women could add another 19 years of life reaching 68 years, before they would need to care for a household member. When we used self-rated health status as an indicator to project healthy life expectancy, the numbers did not differ considerably. In this case, we see immense improvements in healthy life expectancy in recent decades. Over the entire observation period, more than 25 years of life without any subjective health difficulties are added (for the results compare Figure 5). The improvements are less pronounced among women than they are among men, but they are still large. This discrepancy may be partially caused by men’s tendency to report better health than they objectively have [46].

**Figure 6:**
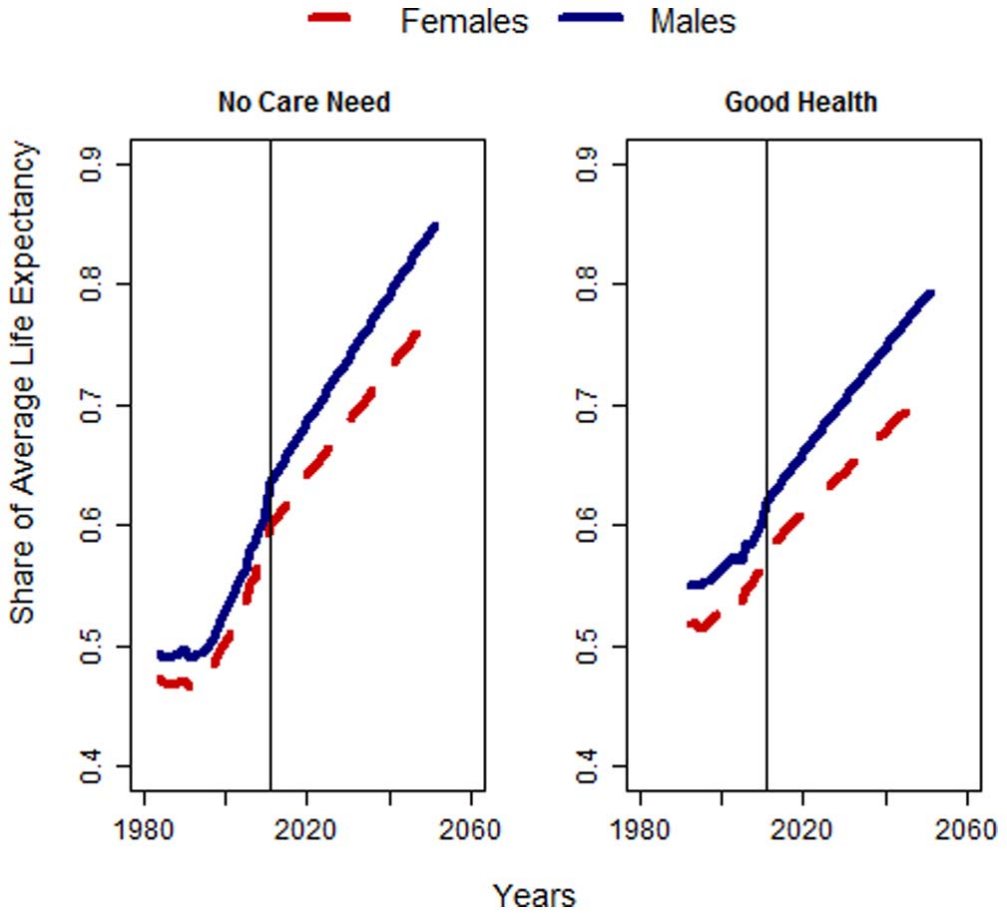
Share of lifetime spend with no care provision for any elderly household member and good health, men and women, Germany 1984–2050. Source: GSOEP 1984–2011, UN population projection.

Figure 6 shows the results in relation to life expectancy. We used data for Germany from the Human Mortality Database for the years 1956 to 2011 and the Lee-Carter-Method to forecast future life expectancy at birth [47]. Both of the indicators show a significant increase in the share of the lifetime spent in good health and with no care need. We estimate that the average man will spend around 80% of his lifetime in good health in 2050, compared to 63% today. The gap between men and women is slightly bigger in this case due to the higher life expectancy among women. In 2050, the share of the lifetime spent in good health is expected to be seven percentage points lower among women than it is among men. Still, the average woman in 2050 will likely spend over 70% of her lifetime in good health, compared to less than 50% in the past and about 60% today. According to our estimates, the onset of care need for spouses or elderly members of the household will be pushed to higher ages. Based on the development of care need since 1984, for the future our results suggest that we will spend the majority of our life time without someone in the household who needs care.

### Life-cycle adjustments? – Demographic change and time use

The ratio of market work, housework, and leisure over the life-cycle and in cross-section is of increasing importance, both between the sexes and across the life-cycle at the individual level. As the population ages and the pressure for women to participate in the labor force grows, this area is expected to undergo significant changes, which will in turn have an impact on the current generational and gender contract. We estimated and studied monetary estimates of income, consumption, and intergenerational transfers obtained by following the standard methodology of the National Transfer Accounts. This process provided us with age-specific economic variables which were also decomposed by gender. The theoretical framework was built upon Samuelson [48], Diamond [49], and Lee [50]. Information about consumption, income, and the age utilization of public expenditures and revenues came from the database for Germany [51]. To estimate production, consumption, and transfers in the household, the Time Use Survey 2001/02 was employed. The observed differences in time use by gender provide information about the division of domestic work within households, and the overall workload of an individual. While working hours in market employment were found to be twice as high for men as they are for women, the reverse was shown for domestic labor. Women were found to perform two-thirds of the domestic services, including general housekeeping tasks, childcare, elderly care, and shopping (Table 2).

**Table 2:** Hours spent on housework per day by gender.

	<30	30–65	65+
Men	1.3	2.8	3.7
Women	2.2	4.8	4.9

Source: Time Use Survey 2001/02, Germany.

Market consumption and labor income, as well as the corresponding difference between the two, or the life-cycle deficit, show a not surprising pattern. We found that men tend to produce twice as much labor income as women beginning in their thirties. As the consumption levels were found to be relatively comparable, the average woman was not shown to have a surplus over her life course. This picture changes dramatically if we include unpaid home production. For almost all of the items, such as cooking, cleaning, or shopping, women have significantly higher values of hours worked. Only from retirement age onward were men shown to increasingly engage in cooking, cleaning, and gardening. We used the specialist replacement method to monetize unpaid housework according to the third-party rule [52], we use the hourly wage of an unskilled worker for all easy household tasks. When we combined market and unpaid home production by age and gender, we found roughly similar life-cycle patterns of consumption and income for both sexes. The monetized results for the lifecycle deficit with and without household production and consumption are shown in Table 3.

**Table 3:** Market and non-market values for consumption, income, and corresponding lifecycle deficit in billion euros, Germany 2003.

	*Men*	*Women*	*Total*
*Consumption*	*725*	*781*	*1506*
*Income*	*814*	*429*	*1243*
*Market Lifecycle Deficit*	*89*	*−352*	*−263*
*Time adjusted consumption*	*1,131*	*1,202*	*2,333*
*Time adjusted income*	*1,124*	*946*	*2,070*
*Time adjusted lifecycle deficit*	*−7*	*−256*	*−263*

Source: own calculations.

The relationship between leisure, market work, and housework is expected to change in the future, assuming the currently observed profiles continue, as is shown in Table 4. At the population level, we found that the amount of time devoted to home production is likely to exceed the time spent performing market work. The amount of time spent on market work as a share of the total time available was only 14.5% in 2010, and is expected to decrease to 11.9% by 2060. The analysis showed that the share of time spent in what we call leisure – i.e., activities other than market work, home production, or sleeping – is set to increase slightly over the study period among the older population. For the average individual (in this synthetic cohort approach), we found that the share of time spent working is projected to be 7.4%, while the share of time spent in leisure activities is likely to account for nearly half of the person’s lifetime. Thus, the aging of the population will lead to an increase in leisure time, if no further adjustments are made.

**Table 4:** Percentage share of activities at the population level, Germany 2010 and 2060.

	*Housework*	*Work*	*Leisure*
*2010*	*20.5*	*14.5*	*65.0*
*2060*	*21.8*	*11.9*	*66.3*

Source: Time Use Survey 2001/02, own calculations.

It would be interesting to estimate a scenario assuming participation rates of Swedish men and women but time transfers for Sweden are not yet available. Still, with a simple ten percentage points increase in female labor force participation we can assume the iso-work amount to stay constant. In response, females could reduce hours in unpaid domestic labor. We find that 47 million hours of work would need to be redistributed on the population level. About 30,000 individuals working on average 30 hours per week would be needed to close the gap. In case the excess housework is split between household members each adult had to increase weekly working hours by about 45 minutes.

## Discussion

In this paper, we addressed the important question of how selected areas of life will be affected as populations grow older and smaller. We used the case of Germany, a country that is at a relatively advanced stage of the demographic transition, to study the potential long-run implications of population aging. In the decades prior to 2040, the consequences of population aging are expected to be largely negative, as the baby boomers reach retirement age and dependency ratios and expenditures for pensions and health care increase. Still, we found evidence that after a period of transition, population aging could have effects that are positive, at least in part.

We have shown the potential effects of having an older and smaller population, such as lower carbon emission levels due to changes in aggregate consumption patterns. Having a higher share of the population with tertiary education could be beneficial for economic growth in the long run, and could compensate, at least in part, for absolute and relative declines in the number of economically active persons. Inherited wealth will have to be split among a smaller number of siblings, which could compensate for an increase in upward public transfers, via pay-as-you-go pension system. Furthermore, individuals are expected to spend longer shares of their lives in good health and engage in leisure activities.

The results only take into account pure demographic changes, as we chose to look at only the changes related to shifts in the population composition, and their impact on different areas of society. Given this limitation, we were unable to predict whether behavioral changes will occur as individuals adapt to an older population structure. We are aware that changing expectations will alter today’s observed profiles.

The higher educational attainment at the population level can be advantageous for economic growth. Whether this development, through presumed increases in productivity, can compensate for the negative long-run effects of the shrinking workforce remains to be seen. The positive returns of education might be smaller in the future, when larger shares of each cohort attain higher levels of educational attainment than it is currently the case. The expected reduction in the levels of carbon emissions due to population decline could be even greater if more environmentally-friendly technologies are adopted. On the other hand, emissions could also stagnate, as shrinking populations might not be able to realize significant CO_2_ reductions. For example, more individuals may be living in smaller or single households that are not efficient in terms of per capita energy use. The higher educational attainment could also be disadvantageous for the level of CO_2_ emissions as individuals with tertiary education tend to travel more. The bequests received by future generations could be significantly lower than our estimates if their parents plan for longer lives and adapt to increasing longevity by using/depleting their capital stock. Moreover, the projected increase in healthy life expectancy is difficult to estimate. The question of whether the time spent in intensive care will really be postponed to the last two years of life, as some studies have proposed [45], [53], remains open. Trends in dementia, for example, are not entirely clear [54], [55]. Furthermore, given the increases in the pension entitlement age in most developed countries, the share of the lifetime spent working could remain constant instead of declining.

Nevertheless, the results show that there are important areas in which population aging and decline could be beneficial. Projecting the behavioral changes in each of these areas is beyond the scope of this article, but this is an obvious topic for future research. Although the results presented refer to Germany, they could be of interest for other regions around the world, including both developed and developing countries. Life expectancy is increasing almost universally, and the health status of the older population is improving. The importance of investments in education is well known, and education levels are increasing in the great majority of countries. Depending on a country’s stage in the demographic transition process, the results from the analyses of bequests and CO_2_ emissions are also generalizable.

Some aspects of population aging are an inevitable consequence of demographic change. In this article, we showed examples of situations where population aging can lead to important opportunities for our societies. We believe that a continued discussion about these issues between social scientists and policy makers will be crucial to leverage the benefits of changing population compositions.
